# Supplementary material for: Ebselen as template for stabilization of A4V mutant dimer for motor neuron disease therapy
Source: Commun Biol. 2020 Mar 5;3:97. doi: 10.1038/s42003-020-0826-3 (PMC7058017; doi:10.1038/s42003-020-0826-3)
Supplement: Supplementary file 1 — Supplementary Information [file 42003_2020_826_MOESM1_ESM.pdf]

Supplementary Information

Title: Ebselen as template for stabilisation of A4V mutant dimer for motor neuron disease therapy

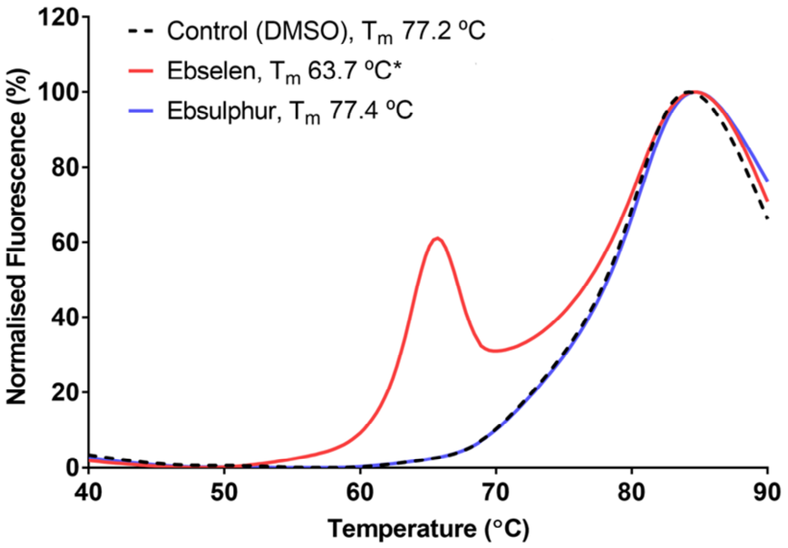

Supplementary figure 1. Melting curves and melting temperatures of C111S SOD1 with and without ebselen or ebsulphur. Figures are means from 2 independent experiments. * indicates the first T_m of the biphasic curve derived by calculating the peak of the first derivative of the raw data.

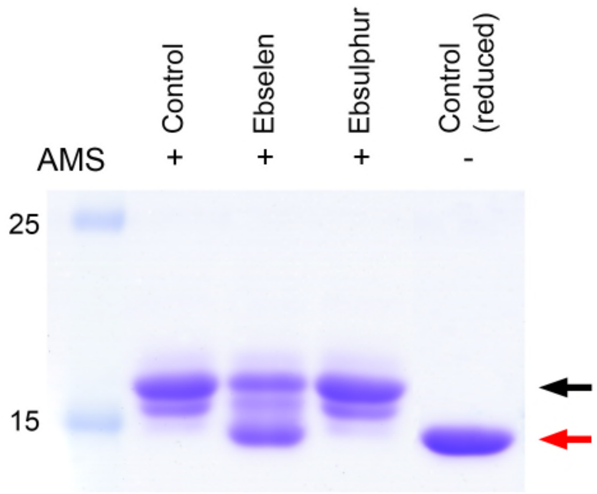

Supplementary figure 2. AMS modification of A4V^{C6S} SOD1. A4V^{C6S}-ebsulphur migrates to the same level as the control indicating Cys111 is not protected by reaction with ebsulphur (black arrow). Conversely, A4V^{C6S}-ebselen shows a strong band equivalent to disulphide reduced and unmodified SOD1 thus indicating Cys111 is extensively protected by ebselen binding (red arrow).

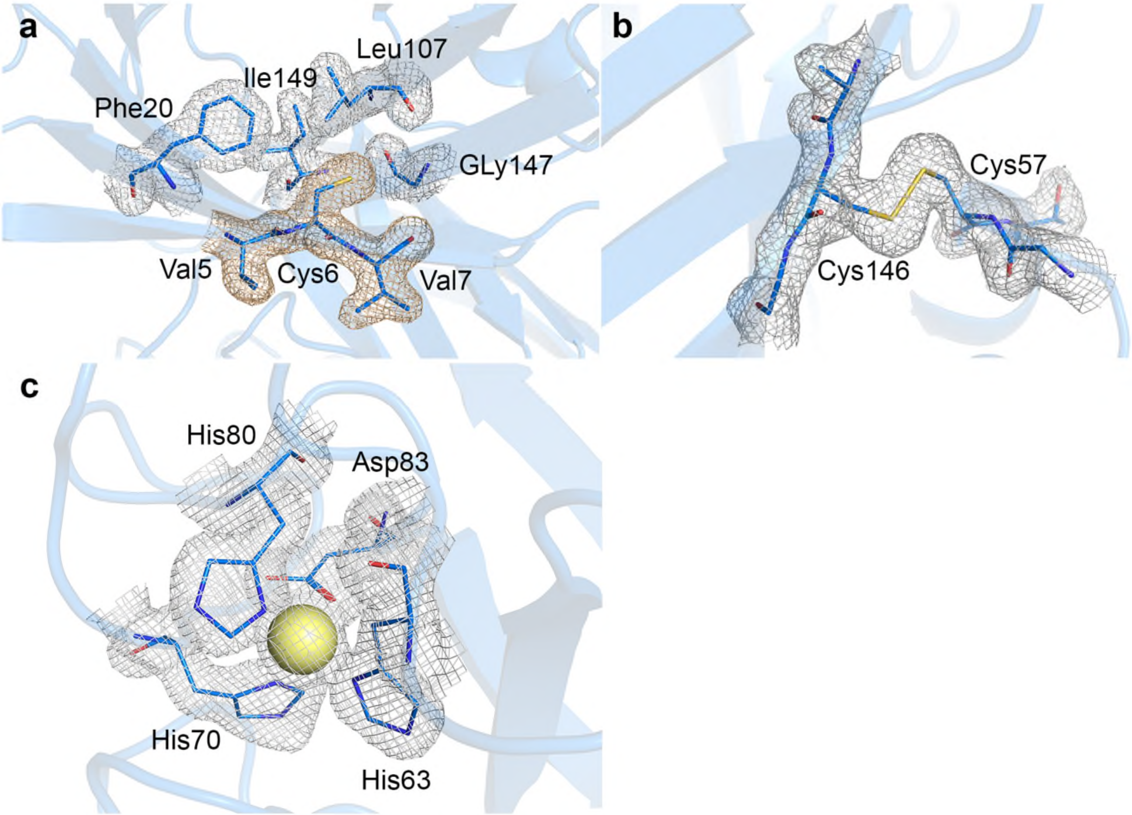

Supplementary figure 3. 2Fo-Fc electron density maps of A4V-ebselen crystal structure contoured at 1 σ. **a** shows Zn (yellow sphere) in the Zn binding site. **b** shows intact free Cys6 facing inside the β-barrel structure and its neighbouring residues. No electron density of any compounds is observed near Cys6. The electron density map of Val5, Cys6 and Val7 is shown in orange. The electron density map of other residues are shown in grey. **c** shows intact disulphide bond between Cys57 and Cys146.

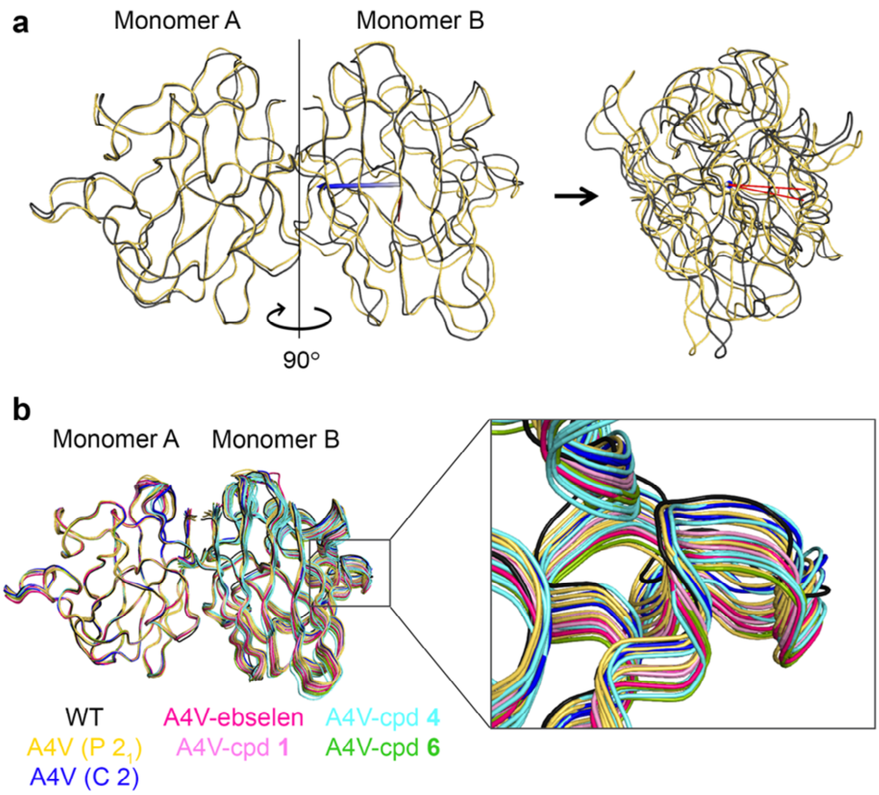

Structures	No. of dimers in the ASU	RMSD (Å)	Angle of rotation (°)	Displacement (Å)
A4V (P 2$_1$)	6	1.22-1.74	4.90-8.28	0.19-0.55
A4V (C 2)	3	0.96-1.30	3.55-5.99	0.23-0.35
A4V-ebselen	3	1.38-1.62	5.96-7.36	0.30-0.64
A4V-cpd **1**	3	1.37-1.66	5.80-7.71	0.25-0.31
A4V-cpd **4**	6	0.93-1.64	3.54-5.22	0.39-0.92
A4V-cpd **6**	3	1.59-1.81	6.45-6.98	0.57-1.01

Supplementary figure 4. Monomer-monomer reorientation. Crystal structures of the wild-type SOD1 structure (PDB code: 2V0A) overlaid with all A4V SOD1 structures (P 2$_1$; PDB code: 1UXM, and C 2) and ligand-conjugated A4V SOD1 structures. The main chain of monomer A of each structure was superposed to demonstrate the orientation of the monomer B. **a** Measurement of the angle of rotation. Only one chain of A4V SOD1 structure is shown, to represent other structures. The blue arrow represents the axis of rotation, which is perpendicular to the dimer interface plane and slightly different among the structures. The two red lines represent the angle of rotation from the wild-type structure. **b** Variation of the dimers in each protein after superposing all molecules in the asymmetric units. The expanded box shows the details of variations at the electrostatic loop which is is the furthest the region in monomer B and allows us to observe the difference among this monomer when monomer A is superposed. The table shows the maximum and the minimum of RMSD, angle of rotation and displacement of each protein compared to the wild-type structure. ASU; asymmetric unit

Supplementary table 1. A4V and ligand-conjugated A4V dimer interface non-covalent bonding interactions.

**Amino acid**	**Group**	**Distance**							**Group**	**Amino acid**
		Wild-type	A4V ($P\ 2_1$)	A4V (C 2)	A4V-ebselen	A4V-cpd **1**	A4V-cpd **4**	A4V-cpd **6**		
**Hydrogen bonding**										
Gly51	Amine (N)	2.80	2.75±0.08	2.76±0.02	2.73±0.06	2.70±0.05	2.71±0.06	2.77±0.04	Carbonyl (O)	Ile151
Ile151	Amine (N)	2.90	2.84±0.13	2.76±0.03	2.79±0.07	2.77±0.07	2.78±0.05	2.71±0.06	Carbonyl (O)	Gly114
**Hydrophobic bonding**										
Ala152, Gln153	Amide	4.64	4.51±0.08	4.48±0.04	4.37±0.01	4.42±0.08	4.53±0.10	4.26±0.07	Phenyl	Phe50
Arg115	Alkyl	5.43	5.29±0.11	5.17±0.04	5.20±0.52	5.36±0.0	5.29±0.09	5.43±0.03	Alkyl	Ile151
Val148	Alkyl	4.33	4.20±0.06	4.20±0.05	4.60±0.67	4.25±0.10	4.30±0.04	4.26±0.05	Alkyl	Val148

The figures are mean ± standard deviation of symmetrical bond distances in all dimer interfaces in the asymmetric unit.

Supplementary table 2. Distance between Cα of the residues at the dimer interface.

**Amino acid**	**Distance**							**Amino acid**
	Wild-type	A4V (P 2_1)	A4V (C 2)	A4V-ebselen	A4V-cpd **1**	A4V-cpd **4**	A4V-cpd **6**	
Phe50	5.30	5.30±0.09	5.35±0.04	5.35±0.07	5.32±0.09	5.26±0.07	5.37±0.04	Ala152
Gly51	3.88	4.01±0.11	3.92±0.05	3.96±0.03	3.94±0.07	3.94±0.08	3.89±0.04	Gly150
Asp52	6.09	6.15±0.05	6.17±0.02	6.19±0.04	6.21±0.09	6.20±0.09	6.27±0.06	Val5
Asn53	6.58	6.29±0.10	6.38±0.03	6.31±0.06	6.26±0.04	6.32±0.26	6.21±0.05	Val7
Thr54	7.11	7.20±0.07	7.17±0.06	6.96±0.10	7.00±0.06	6.86±0.24	6.84±0.10	Ile17
Ala55	11.78	12.02±0.18	12.27±0.11	11.90±0.12	11.84±0.11	11.80±0.26	12.09±0.55	Gly16
Gly56	12.45	12.19±0.21	12.23±0.12	11.93±0.15	11.66±0.14	12.20±0.17	11.79±0.09	lys9
Gly114	4.19	4.10±0.07	4.06±0.04	4.05±0.07	3.99±0.04	4.15±0.03	3.88±0.04	Gly150
Val148	6.11	6.19±0.08	6.15±0.02	6.07±0.03	6.01±0.03	6.01±0.09	5.82±0.07	Val148
Gly108	10.91	11.91±0.36	10.42±0.03	12.06±0.17	11.70±0.08	12.93±1.01	13.05±0.23	Gly108
Asp109	15.57	16.51±0.29	13.67±0.71	16.25±0.43	16.67±0.11	16.27±0.45	16.85±0.39	Asp109
His110	19.39	19.78±0.13	17.87±0.59	20.21±0.11	20.23±0.16	19.31±0.32	20.33±0.20	His110
Cys111	13.39	13.47±0.11	12.63±0.20	13.83±0.09	13.89±0.17	13.49±0.37	13.89±0.18	Cys111
Ile112	14.02	13.93±0.09	13.75±0.07	14.27±0.06	14.22±0.04	14.35±0.18	14.55±0.09	Ile112
Ile113	7.41	7.21±0.11	6.95±0.05	7.26±0.02	7.28±0.03	7.52±0.14	7.48±0.09	Ile113
Gly114	4.53	4.58±0.08	4.57±0.02	4.77±0.03	4.79±0.03	4.48±0.09	4.87±0.02	Gly114

The figures are mean ± standard deviation of the distances of all dimer interfaces in the asymmetric unit.
Highlights are the distances of the residues in loopVI between two monomers of the C2 A4V SOD1 structures.

Supplementary Method

The synthesis of the ebselen derivatives all follow a similar route. For targets **1** and **2**, 2-iodobenzoic acid was converted to an acid chloride intermediate which was telescoped into the into a solution of the aniline to allow amide bond formation in high yields. The amide was carried through into the next step without the need of purification. The target ebselen analogues were prepared by a Cu(I) catalysed ring closing reaction of the requisite iodo-amide intermediate and selenium powder.

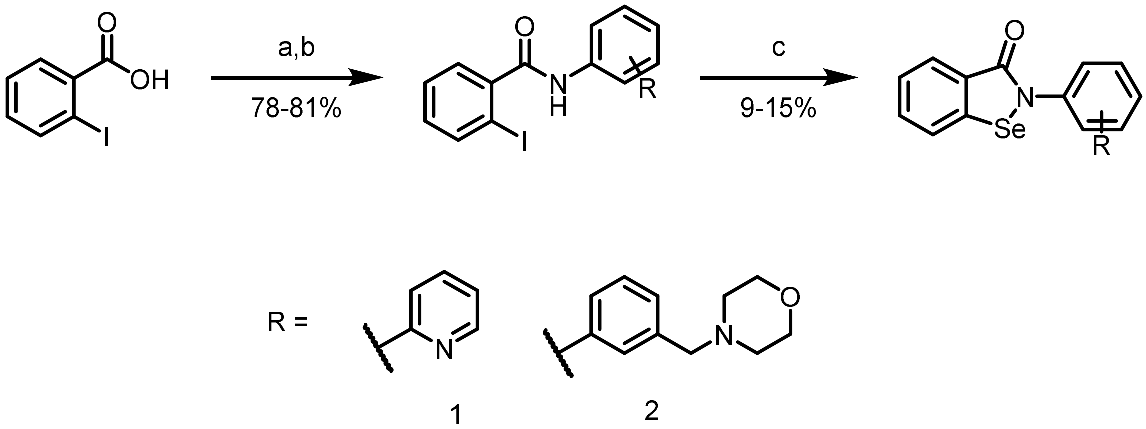

Scheme S1: a) $C_2O_2Cl_2$, cat. DMF, DCM, 1-2 hours; b) aniline derivative, NEt_3, DCM, overnight; c) CuI, 1,10-phenanthroline, Se powder, K_2CO_3, DMF, 110°C.

Compounds **6-10** contain biaryl rings on the right hand side (RHS) of the compound template. In order to synthesis these biaryl ring systems, Suzuki chemistry was employed in high yields as shown in Scheme S2.

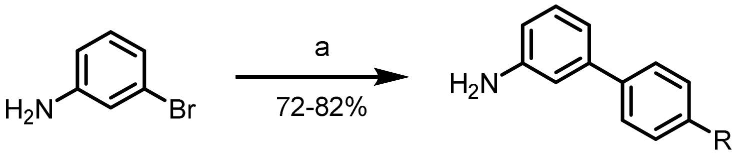

Scheme S2: a) R-B(OH)$_2$, Pd(PPh$_3$)$_4$, K_2CO_3, THF/H_2O, 80°C, overnight.

The starting boronic acids for compounds **9-10** were not available and therefore were synthesised in by S_N2 chemistry to afford the desired boronic acids in good to excellent yields. These were subjected to the Suzuki conditions to afford the biaryl aniline intermediates as shown in Scheme S3.

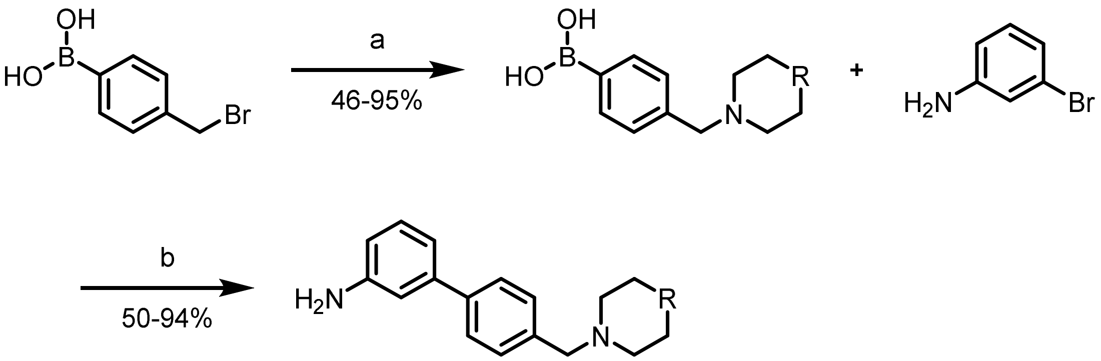

Scheme S3: a) amine, DIPEA, THF, overnight; b) R-B$(OH)_2$, Pd$(PPh_3)_4$, K_2CO_3, THF/H_2O, 80°C, overnight.

The amide intermediates were allowed to react under the same conditions previously shown in Scheme 1. The newly formed biaryl amine underwent amide formation with the acid chloride intermediate of 2-iodobenzoic acid. Yields varied greatly depending on the amine derivatives used to afford the analogues in Scheme S4. Reaction of the amides under Cu(I)I catalysed ring closing conditions gave the desired compounds **6-10**, again in low yields.

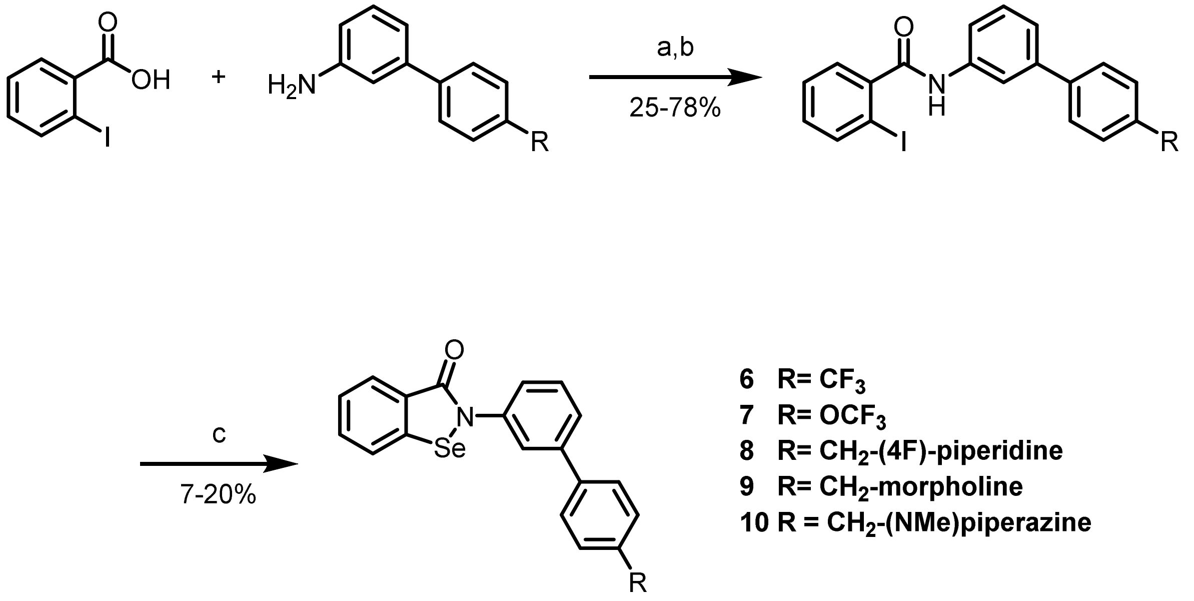

Scheme S4: a) $C_2O_2Cl_2$, cat. DMF, DCM, 1-2 hours; b) aniline derivative, NEt_3, DCM, overnight; c) CuI, 1,10-phenanthroline, Se powder, K_2CO_3, DMF, 110°C.

Compounds **3-5** contain a methylene linker between the RHS phenyl ring and the selenium-containing core. The synthesis of these analogues follows the same synthetic route as the other analogues, shown in Schemes **1** & **4**. However benzylamine derivatives are coupled with the acid chloride of 2-iodobenzoic acid, shown in Scheme S5, in excellent yields for amide intermediates **x**. Cu(I)I catalysed ring closing conditions are employed again to yield the desired compounds in low yields.

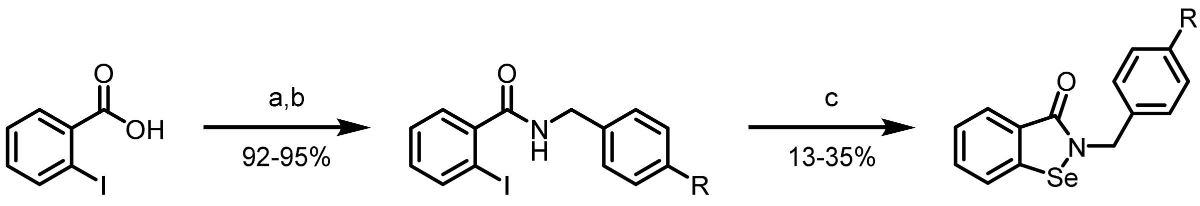

3 R= H
4 R= Cl
5 R= OMe

Scheme S5: a) $C_2O_2Cl_2$, cat. DMF, DCM, 1-2 hours; b) benzylamine derivative, NEt_3, DCM, overnight; c) CuI, 1,10-phenanthroline, Se powder, K_2CO_3, DMF, 110°C.

General Procedure 1: Amide preparation

Step 1: To a stirring solution of the required carboxylic acid (1 eq) in anhydrous DCM (0.2M) was added oxalyl chloride (2 eq) and catalytic DMF (3-4 drops). The resulting reaction mixture was stirred at room temperature for 1.5 hours. The reaction mixture was then concentrated under reduced pressure and the resulting acid chloride intermediate dried thoroughly under high-vacuum. The acid chloride was re-dissolved in anhydrous DCM (0.2M), followed by the selected amine (2 eq) and NEt_3 (2-3 eq). The reaction mixture was left to stir overnight or until acid chloride was completely consumed under an N_2 environment. The reaction mixture was diluted with DCM and washed with distalled water, 1M HCl, sat. $NaHCO_3$ and brine. The organic phase was dried over anhydrous $MgSO_4$, filtered and concentrated under reduced pressure. The crude products were purified by FCC (flash chromatography) if required.

General procedure 2: Benzisoselenazolone formation via CuI ring closure

A solution of copper iodide (0.2-1.0 eq) and 1,10-phenanthroline (0.2-1.0 eq) in DMF (30 mL/g) was stirred under nitrogen for 15 minutes (dark orange/brown solution). The required amide (1 eq), selenium (1.2 eq) and potassium carbonate (1.5 eq) were then added before heating to 110°C for 36 hours under a nitrogen atmosphere. The reaction mixture was then stirred in brine for 3 hours before extracting with ethyl acetate (3 x 30mL). The combined organic extracts were then dried over anhydrous $MgSO_4$, filtered and concentrated under reduced pressure. The crude products were purified by flash chromatography.

General Procedure 3: Suzuki couplings

To a mixture of THF (1.5M) and water (1.5M) was added 3-bromoaniline (3 eq) and potassium carbonate (3 eq) the flask was evacuated and backfilled with N_2. The selected boronic acid (1 eq) was added, the flask evacuated and backfilled with N_2. Finally palladium tetrakis (2.5 mol%) was added and the reaction vessel was evacuated and backfilled with N_2 before refluxing at 80 °C overnight. The reaction mixture was diluted with brine and product extracted into ethyl acetate. The Organic phases were dried with $MgSO_4$ and product purified by FCC.

General Procedure 4: Preparation of amine coupled aryl side chains

To (4-(bromomethyl)phenyl)boronic acid (1 eq) was added THF (0.5M), DIPEA (1.2 eq) and selected amine (1.2-1.5 eq). The reaction was stirred at room temperature under a N_2 atmosphere overnight. The reaction was diluted with brine and extracted into ethyl acetate (x 3), dried with $MgSO_4$ and solvents removed in *vacuo* to yield the desired product. No further purification.

General Procedure 5– Amide coupling conditions for final products

To a flask at room temperature was added 2-(3-oxobenzo[d]isothiazol-2(3H)-yl)acetic acid (1.0 eq), DMF (5 ml) EDC.HCl (1.2 eq) and pyridine (2.0 eq). The reaction mixture was left to stir for 15 minutes followed by the addition of the desired amine (1.0 eq) and further pyridine (2.0 eq.) and the reaction stirred overnight at room temperature. The reaction was then diluted with ethyl acetate, acidified with 1 M HCl, basified with 1 M NaOH, washed with water, brine, dried over $MgSO_4$ and concentrated to yield the crude product.

General Procedure 6 – Amide coupling to give 2-(1isoindolin-2-yl)acetamide Derivatives

To a flask at room temperature was added 2-(1-oxoisoindolin-2-yl)acetic acid derivative (1.0 eq), DMF, EDC.HCl (1.5 eq), pyridine (2.5 eq), and the desired amine (1.0 eq) and the reaction stirred at room temperature overnight. The reaction was then diluted with ethyl acetate, acidified with 1M HCl, basified 1M NaOH, washed with water, brine, dried over $MgSO_4$, concentrated to yield the crude product.

General Procedure 7 – Alkylation of the BIZT core using benzyl bromides

To a flask at room temperature was added benzo[*d*]isothiazol-3(2*H*)-one (1eq), THF, potassium carbonate (2.5 eq) and the desired benzyl bromide (2.0 eq), and the reaction stirred at room temperature overnight. The reaction was then concentrated, diluted with ethyl acetate, washed with a saturated $NaHCO_3$ solution, water, brine, dried over $MgSO_4$, concentrated to yield the crude product.

HPLC

Flow rate 1 ml/min for 15 minutes using MeCN/Water with compounds dissolved in methanol. UV detector recorded signals at 254 nm. Method: min, gradient: 2% MeCN hold to 1 min, 2-98% MeCN in 11 min, then hold at 98% MeCN to 15 min.

2-Iodo-*N*-(pyridin-2-yl)benzamide

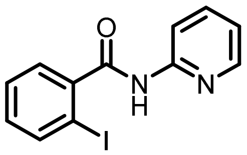

General procedure 1 was followed using 2-iodobenoic acid and 2-aminopyridine (omitting the acid wash from the work up due to the presence of pyridine) to give the title compound as a yellow oil (1.06 g, 81% yield): ^{1}H NMR (400 MHz, $CDCl_3$) δ =8.47 (dd, *J* = 5, 2 Hz, 1H), 7.80 – 7.74 (m, 2H), 7.60 – 7.56 (m, 2H), 7.31 (t, *J* = 8 Hz, 2H), 7.00 (td, *J* = 8, 2 Hz, 2H). ^{13}C NMR (101 MHz, $CDCl_3$) δ = 171.0, 152.1, 149.6, 147.9, 140.8, 140.5, 140.3, 138.9, 138.3, 131.9, 131.5, 129.4, 128.5, 128.0, 123.5, 123.4, 120.4, 114.5, 93.5. HRMS (CI, *m/z*) calculated for $C_{12}H_{10}IN_2O$ [M+H]+ 324.9838, found 324.9843.

2-(Pyridin-2-yl)benzo[*d*][1,2]selenazol-3(2*H*)-one (1)[1]

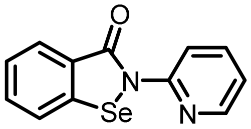

General procedure 2 was followed using 2-iodo-*N*-(pyridin-2-yl)benzamide to give the title compound as an off-white solid (80 mg, 9% yield). ^{1}H NMR (400 MHz, $CDCl_3$) δ = 8.71 (d, *J* = 8 Hz, 1H), 8.34 (dd, *J* = 5, 1 Hz, 1H), 8.07 (d, *J* = 8 Hz, 1H), 7.80 – 7.73 (m, 1H), 7.68 – 7.60 (m, 2H), 7.42 (ddd, *J* = 8, 7, 2 Hz, 1H), 7.10 (ddd, *J* = 7, 5, 1 Hz, 1H). ^{13}C NMR (101 MHz, $CDCl_3$) δ = 165.1, 151.6, 147.6, 138.9, 138.4, 132.9, 130.9, 126.2, 123.9, 120.9, 114.5.

HRMS (CI, *m/z*) calculated for $C_{12}H_9N_2OSe$ [M+H]+ 276.9880, found 276.39876. IR (neat) ν_{max}/cm^{-1} 2921(w, aromatic C-H), 2851 (m, C-H aliphatic), 1606 (s, C=O amide), 1444 (s, C-C aromatic), 1427 (s, C-C aromatic), 1308 (m, C-N amine aromatic) cm^{-1}. Melting Point: 233-234°C.

4-(3-Nitrobenzyl)morpholine

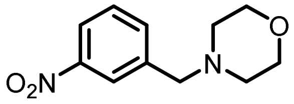

General procedure 4 was followed using 1-(bromomethyl)-3-nitrobenzene and morpholine to give the target as a white solid (2.04 g, 99 %). ^{1}H NMR (400 MHz, $CDCl_3$) δ = 8.22 (s, 1H), 8.12 (d, *J* 8.2 Hz, 1H), 7.68 (d, *J* 7.6 Hz, 1H), 7.49 (apparent t, *J* 7.9 Hz, 1H), 3.76 – 3.69 (m apparent t, 4H), 2.50 – 2.42 (apparent t, 4H). ^{13}C NMR (101 MHz, $CDCl_3$) δ = 148.5, 140.4, 135.2, 129.4, 123.9, 122.5, 67.0, 62.5, 53.7. HRMS (CI, *m/z*) calculated for $C_{11}H_{15}N_2O_3$ $[M+H]^+$ requires 223.1077. Found 223.1086.

3-(Morpholinomethyl)aniline

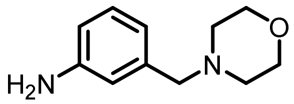

To 4-(3-nitrobenzyl)morpholine (2.04 g, 9.16 mmol, 1 eq) was added ethanol (20 cm^3), acetic acid (20 cm^3) and iron powder (2.56 g, 45.79 mmol, 5 eq). The reaction was refluxed overnight under an N_2 atmosphere. Reaction mixture was filtered through celite, and washed with EtOAc (50 cm^3), solvents were removed in *vacuo*. Water (100 cm^3) was added and the product extracted into EtOAc (3 x 100 cm^3), organics washed with saturated $HNaCO_3$ (100 cm^3), water (100 cm^3), brine (100 cm^3) and dried over $MgSO_4$. Solvents removed in *vacuo* to give the product as a brown oil (1.68 g, 96 %). ^{1}H NMR (400 MHz, $CDCl_3$) δ = 7.09 (apparent t, 1H), 6.72 – 6.68 (m, 2H), 6.60 – 6.56 (m, 1H), 3.77 – 3.59 (m, 6H), 3.40 (s, 2H), 2.48 – 2.38 (m, 4H). ^{13}C NMR (101 MHz, $CDCl_3$) δ = 146.5, 139.1, 129.2, 119.7, 115.9, 114.1, 67.1, 63.6, 53.8. HRMS (CI, *m/z*) calculated for $C_{11}H_{17}N_2O$ $[M+H]^+$ requires 193.1335. Found 193.1340.

2-Iodo-*N*-(3-(morpholinomethyl)phenyl)benzamide

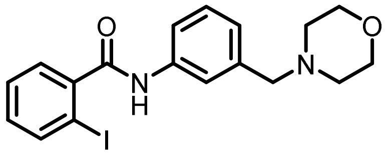

General procedure 1 was followed using 3-(morpholinomethyl)aniline and 2-iodobenzoic acid. Note HCl not used in workup to give the amide as a white to yellow semi solid (1.71 g, 78 %). Compound used without further purification. ^{1}H NMR (400 MHz, $CDCl_3$) δ = 7.90 (d, *J* 7.8 Hz, 1H), 7.65 (s, 1H), 7.59 – 7.55 (m, 2H), 7.50 (dd, *J* 7.6, 1.5 Hz, 1H), 7.44 – 7.39 (m, 1H), 7.32 (apparent t, 1H), 7.17 – 7.11 (m, 2H), 3.72 – 3.66 (m, 4H), 3.50 (s, 2H), 2.50 – 2.40 (m, 4H). ^{13}C NMR (101 MHz, $CDCl_3$) δ = (101 MHz, $CDCl_3$) 167.4, 142.2, 140.2, 139.2, 137.7, 131.6, 129.1, 128.6, 128.5, 125.7, 120.7, 119.1, 92.5, 67.1, 63.3, 53.7. HRMS (ES+, *m/z*) calculated for $C_{18}H_{20}IN_2O_2$ $[M+H]^+$ requires 423.0564. Found 423.0568.

2-(3-(Morpholinomethyl)phenyl)benzo[*d*][1,2]selenazol-3(2*H*)-one (2)

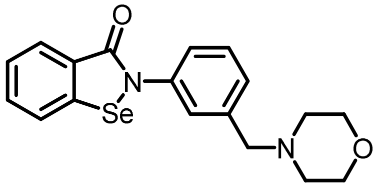

General procedure 2 was followed using 2-iodo-*N*-(3-(morpholinomethyl)phenyl)benzamide. The crude product was purified by FCC (100 % EtOAc) and the residue was recrystallised from ethyl acetate to give the target as a white solid (0.088 g, 15 %). ^{1}H NMR (400 MHz, $CDCl_3$) δ = 8.11 (d, *J* 7.9 Hz, 1H), 7.69 – 7.62 (m, 3H), 7.53 – 7.45 (m, 2H), 7.38 (t, *J* 7.8 Hz, 1H), 7.27 – 7.23 (m, 1H), 3.76 – 3.68 (apparent t, 4H), 3.53 (s, 2H), 2.47 (br s, 4H). ^{13}C NMR (101 MHz, $CDCl_3$) δ = 165.8, 139.6, 139.2, 137.7, 132.7, 129.5, 129.3, 127.7, 127.6, 126.7, 126.1, 124.2, 123.9, 67.2, 63.1, 53.7. HRMS (ES+, *m/z*) calculated for $C_{18}H_{19}N_2O_2Se$ $[M+H]^+$ requires 375.0606. Found 375.0615. Elemental analysis calculated for $C_{18}H_{18}N_2O_2Se$ requires, C, 57.91; H, 4.86; N, 7.50, found, C, 57.77; H, 4.79; N, 7.42. IR (neat): ν_{max}/cm^{-1}:

3087 (w), 3054 (w), 2972 (w), 2858 (w), 2801 (w), 1590 (s), 1561 (s), 1442 (s), 1330 (s), 1087 (s). Melting Point:: 179 – 180 (EtOAc).

N-Benzyl-2-iodobenzamide[2]

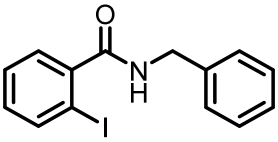

General procedure 1 was followed using 2-iodobenzoyl chloride and benzylamine to give the title compound as a white solid (623 mg, >95% yield): ^{1}H NMR (400 MHz, $CDCl_3$) δ = 7.84 (dd, *J* = 8, 1 Hz, 1H), 7.44 – 7.28 (m, 7H), 7.09 (td, *J* = 8, 2 Hz, 1H), 6.05 (br s, 1H), 4.64 (d, *J* = 6 Hz, 2H). ^{13}C NMR (101 MHz, $CDCl_3$) δ = 169.3, 142.2, 140.1, 137.7, 131.3, 128.9, 128.4, 128.3, 127.9, 92.6, 44.4. HRMS (CI, *m/z*) calculated for $C_{14}H_{13}INO$ [M+H]+ 338.0042, found 338.0047.

2-Benzylbenzo[*d*][1,2]selenazol-3(2*H*)-one(3)[3]

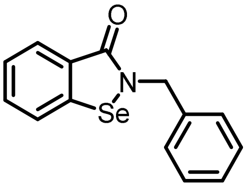

General procedure 2 was followed using *N*-benzyl-2-iodobenzamide to give the title compound as a white solid (185 mg, 35% yield). ^{1}H NMR (400 MHz, $CDCl_3$) δ = 8.08 (d, *J* = 8 Hz, 1H), 7.59 – 7.55 (m, 2H), 7.45 – 7.40 (m, 1H), 7.39 – 7.31 (m, 5H), 5.04 – 5.00 (s, 2H). ^{13}C NMR (101 MHz, $CDCl_3$) δ = 167.3, 138.2, 137.4, 132.2, 129.1, 129.0, 128.7, 128.5, 126.4, 124.1, 48.8. HRMS (CI, *m/z*) calculated for $C_{14}H_{12}NOSe$ [M+H]+ 290.0084, found 290.0086. IR (neat) ν_{max}/cm^{-1} 3033 (w, C-H aromatic), 2980 (w, C-H aliphatic), 1624 (s, C=O amide), 1556 (m, C-C aromatic), 1454 (m, C-C aromatic), 1440 (m, C-H aliphatic) cm^{-1}. Melting Point: 124-126°C.

N-(4-Chlorobenzyl)-2-iodobenzamide[4]

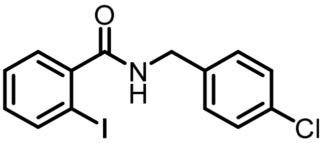

General procedure 1 was followed using 2-iodobenzoic acid and 4-chlorobenzylamine to give the title compound as a white solid (644 mg, 92% yield): 1H NMR (400 MHz, $CDCl_3$) δ = 7.86 (d, *J* = 8 Hz, 1H), 7.42 – 7.30 (m, 6H), 7.10 (td, *J* = 8, 2 Hz, 1H), 6.07 (s, 1H), 4.61 (d, *J* = 6 Hz, 1H). ^{13}C NMR (101 MHz, $CDCl_3$) δ = 169.4, 142.0, 140.1, 136.3, 133.7, 131.5, 129.7, 129.1, 128.5, 128.4, 92.5, 43.6. HRMS (CI, *m/z*) calculated for $C_{14}H_{12}ClINO$ [M+H]+ 371.9652, found 371.9657.

2-(4-Chlorobenzyl)benzo[*d*][1,2]selenazol-3(2*H*)-one (4)

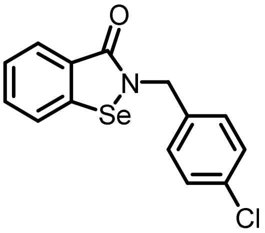

General procedure 2 was followed using *N*-(4-chlorobenzyl)-2-iodobenzamide to give the title compound as a white solid (75 mg, 13% yield). ^{1}H NMR (400 MHz, $CDCl_3$) δ = 8.07 (d, *J* = 8 Hz, 1H), 7.59 (d, *J* = 4 Hz, 2H), 7.46 – 7.41 (m, 1H), 7.35 – 7.27 (m, 4H), 4.98 (s, 2H). ^{13}C NMR (101 MHz, $CDCl_3$) δ = 167.4, 166.6, 138.0, 135.8, 134.4, 132.3, 129.9, 129.4, 129.2, 129.1, 126.5, 124.2, 48.0. HRMS (CI, *m/z*) calculated for $C_{14}H_{11}ClNOSe$ [M+H]+ 323.9694, found 323.9689. Elemental analysis calculated for $C_{14}H_{10}ClNOSe$: C, 52.12; H, 3.12; N, 4.34. Found: C, 52.22; H, 3.07; N, 4.21. IR (neat) ν_{max}/cm^{-1} 3052 (m, C-H aromatic), 2908 (m, C-H aliphatic), 1634 (s, C=O amide), 1586 (s, C-C aromatic), 1443 (m, C-H aliphatic) cm^{-1}. Melting Point: 118-120°C.

2-Iodo-*N*-(4-methoxybenzyl)benzamide

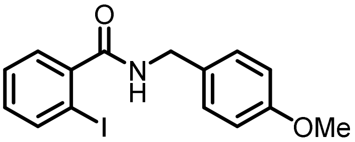

General procedure 1 was followed using 2-iodobenzoic acid and 4-methoxybenzylamine to Give the title compound as a yellow solid (679 mg, >95% yield): ^{1}H NMR (400 MHz, $CDCl_3$) δ = 7.84 (d, *J* = 8 Hz, 1H), 7.41 – 7.36 (m, 1H), 7.36 (d, *J* = 7 Hz, 1H), 7.32 (d, *J* = 9 Hz, 2H), 7.08 (td, *J* = 8, 2 Hz, 1H), 6.88 (d, *J* = 9 Hz, 2H), 6.01 (s, 1H), 4.56 (d, *J* = 6 Hz, 2H), 3.80 (s, 3H). ^{13}C NMR (101 MHz, $CDCl_3$) δ = 168.9, 159.0, 141.9, 139.7, 130.9, 129.4, 129.3, 128.1, 127.9, 113.9, 92.3, 55.1, 43.5. HRMS (CI, *m/z*) calculated for $C_{15}H_{15}INO_2$ [M+H]+ 368.0147, found 368.0156.

2-(4-Methoxybenzyl)benzo[*d*][1,2]selenazol-3(2*H*)-one (5)[5]

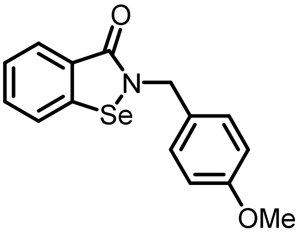

General procedure 2 was followed using 2-iodo-*N*-(4-methoxybenzyl)benzamide to give the title compound as a pale yellow solid (117 mg, 21% yield). ^{1}H NMR (400 MHz, $CDCl_3$) δ = 8.07 (d, *J* = 8 Hz, 1H), 7.55 (d, *J* = 4 Hz, 2H), 7.44 – 7.39 (m, 1H), 7.30 (d, *J* = 9 Hz, 2H), 6.89 (d, *J* = 9 Hz, 2H), 4.94 (s, 2H), 3.81 (s, 3H). ^{13}C NMR (101 MHz, $CDCl_3$) δ = 167.2, 159.9, 138.3, 132.0, 130.3, 129.5, 129.0, 127.9, 126.3, 124.1, 114.3, 55.4, 48.4. HRMS (CI, *m/z*) calculated for $C_{15}H_{14}NO_2Se$ [M+H]+ 320.0190, found 320.0190. Elemental analysis calculated for $C_{15}H_{13}NO_2Se$: C, 56.61; H, 4.12; N, 4.40. Found: C, 56.83; H, 4.09; N, 4.45. IR (neat) ν_{max}/cm^{-1} 3033 (w, C-H aromatic), 2980 (w, C-H aliphatic), 1586 (s, C=O amide), 1557 (m, C-C aromatic), 1440 (m, C-H aliphatic), 1244 (s, C-O) cm^{-1}. Melting Point: 128-130°C.

4'-(Trifluoromethyl)-[1,1'-biphenyl]-3-amine

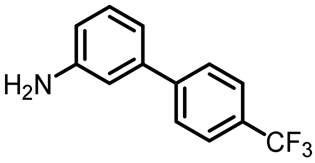

General procedure 3 was followed using (4-(Trifluoromethyl)phenyl)boronic acid. The crude mixture was purified by FCC (20 % EtOAc in Hexane) to give the title compound as a white solid (1.58 g, 75 %).^{1}H NMR (400 MHz, $CDCl_3$) δ = 7.69 – 7.62 (m, 4H), 7.25 (apparent t, 1H), 6.98 (d, *J* 7.6 Hz, 1H), 6.89 (s, 1H), 6.72 (dd, *J* 7.9, 1.8 Hz, 1H), 3.76 (br s, 2H). ^{13}C NMR (101 MHz, $CDCl_3$) δ = 146.9, 144.9, 141.0, 129.9, 129.3 (q, *J* 32.4 Hz), 127.4, 125.6 (q, *J* 3.8 Hz), 124.4 (q, *J* 271.8 Hz), 117.7, 114.9, 113.9. HRMS (CI, *m/z*) calculated for $C_{13}H_{11}F_3N$ $[M+H]^+$ requires 238.0838. Found 238.0845.

2-Iodo-*N*-(4'-(trifluoromethyl)-[1,1'-biphenyl]-3-yl)benzamide

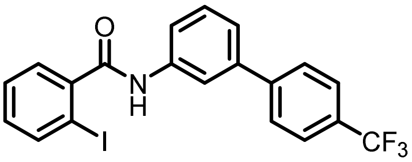

General procedure 1 was followed using 4'-(trifluoromethyl)-[1,1'-biphenyl]-3-amine and 2-iodobenzoic acid to give the title compound as a white solid (1.38 g, 49 %). Compound used in the next step without further purification. ^{1}H NMR (400 MHz, $CDCl_3$) δ = 7.97 (s, 1H), 7.92 (d, *J* 7.9 Hz, 1H), 7.75 – 7.67 (m, 4H), 7.65 – 7.57 (m, 2H), 7.56 – 7.52 (m, 1H), 7.50 – 7.38 (m, 3H), 7.16 (apparent td, 1H). ^{13}C NMR (101 MHz, $CDCl_3$) δ = 167.5, 144.2, 142.1, 141.0, 140.3, 138.3, 132.3 – 131.9 (m), 129.9, 129.8 (q, *J* 33.0 Hz), 128.7, 128.6, 127.7, 125.9 (q, *J* 3.8 Hz), 124.4 (q, *J* 272.1 Hz), 123.9, 119.8, 119.1, 92.5. ^{19}F NMR (375 MHz, $CDCl_3$) δ = -62.4 (3F, s). HRMS (ES+, *m/z*) calculated for $C_{20}H_{13}F_3INNaO$ $[M+Na]^+$ requires 489.9886. Found 489.9890.

2-(4'-(Trifluoromethyl)-[1,1'-biphenyl]-3-yl)benzo[*d*][1,2]selenazol-3(2*H*)-one (6)

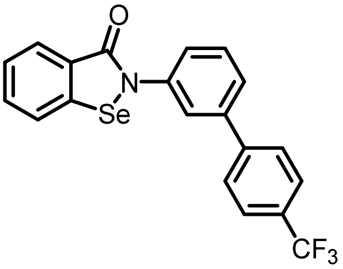

General procedure 2 was followed using 2-iodo-*N*-(4'-(trifluoromethyl)-[1,1'-biphenyl]-3-yl)benzamide. The crude product was purified by FCC (20 % EtOAc in hexane) and the residue was recrystallised from ethyl acetate to give the title compound as white crystals (0.042 g, 7 %). ^{1}H NMR (400 MHz, $CDCl_3$) δ = 8.14 (d, *J* 7.8 Hz, 1H), 7.94 (s, 1H), 7.76 – 7.65 (m, 6H), 7.62 (br d, *J* 7.3 Hz, 1H), 7.58 – 7.45 (m, 3H). ^{13}C NMR (101 MHz, $CDCl_3$) δ = 166.0, 143.9, 141.2, 140.0, 137.7, 132.9, 130.1, 129.8, 129.6, 127.8, 127.6, 126.9, 126.0 (q,

J 3.7 Hz), 125.7, 125.1, 124.5, 123.9. ^{19}F NMR (375 MHz, $CDCl_3$) δ = -62.5 (3F, s). HRMS (ES+, *m/z*) calculated for $C_{20}H_{12}F_3NNaOSe$ $[M+Na]^+$ requires 441.9928. Found 441.9929. Elemental analysis calculated for $C_{20}H_{12}F_3NOSe$ requires C, 57.43; H, 2.89; N, 3.35, found C, 57.46; H, 2.83; N, 3.28. IR (neat): ν_{max}/cm^{-1}: 3088 (w), 3052 (w), 1599 (s), 1563 (s), 1485 (m), 1326 (s), 1305 (s), 1125 (s), 1108 (s). Melting Point: 223 – 224 °C (EtOAc).

4'-(Trifluoromethoxy)-[1,1'-biphenyl]-3-amine

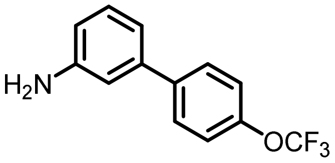

General procedure 3 was followed using (4-(Trifluoromethoxy)phenyl)boronic acid. The crude mixture was purified by FCC (20 % EtOAc in Hex) to give the title compound as a brown oil (0.30 g, 82 %). ^{1}H NMR (400 MHz, $CDCl_3$) δ = 7.58 – 7.54 (m, 2H), 7.28 – 7.19 (m, 3H), 6.94 (ddd, J 7.6, 1.5, 0.9 Hz, 1H), 6.86 (apparent t, 1H), 6.69 (ddd, J 7.9, 2.3, 0.8 Hz, 1H), 3.75 (br s, 2H). ^{13}C NMR (101 MHz, $CDCl_3$) δ = 148.7 (q, J 1.6 Hz), 147.0, 141.2, 140.3, 130.0, 128.5, 121.2, 120.7 (q, J 257.1 Hz), 117.7, 114.6, 113.9. ^{19}F NMR (375 MHz, $CDCl_3$) δ = -57.8 (3F, s). HRMS (CI, *m/z*) calculated for $C_{13}H_{11}F_3NO$ $[M+H]^+$ requires 254.0787. Found 254.0789.

2-Iodo-*N*-(4'-(trifluoromethoxy)-[1,1'-biphenyl]-3-yl)benzamide

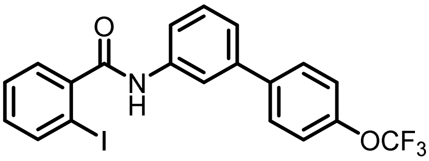

General procedure 1 was followed using 4'-(trifluoromethoxy)-[1,1'-biphenyl]-3-amine and 2-iodobenzoic acid to give the title compound as an off white to yellow powder (0.15 g, 35 %). Compound used in the next step without further purification. ^{1}H NMR (400 MHz, $CDCl_3$) δ = 7.95 – 7.88 (m, 2H), 7.66 – 7.57 (m, 4H), 7.54 (dd, J 7.6, 1.3 Hz, 1H), 7.48 – 7.41 (m, 2H), 7.37 (d, J 7.7 Hz, 1H), 7.28 (d, J 8.2 Hz, 2H), 7.16 (apparent td, 1H). ^{13}C NMR (101 MHz, $CDCl_3$) δ = 167.4, 149.0, 142.1, 141.1, 140.3, 139.5, 138.2, 131.8, 129.8, 128.8, 128.6, 123.8,

121.4, 119.4, 119.0, 92.5. HRMS (ES+, *m/z*) calculated for $C_{20}H_{13}F_3INNaO_2$ $[M+Na]^+$ requires 505.9835. Found 505.9847.

2-(4'-(Trifluoromethoxy)-[1,1'-biphenyl]-3-yl)benzo[*d*][1,2]selenazol-3(2*H*)-one (7)

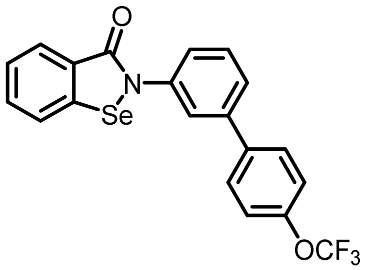

General procedure 2 was followed using 2-iodo-*N*-(4'-(trifluoromethoxy)-[1,1'-biphenyl]-3-yl)benzamide. The crude product was purified by FCC (25 % EtOAc in hexane) and the residue was recrystallised from ethyl acetate to give the title compound as a static white solid (0.024 g, 20 %). ^{1}H NMR (400 MHz, $CDCl_3$) δ = 8.14 (d, *J* 7.8 Hz, 1H), 7.89 (apparent t, *J* 1.8 Hz, 1H), 7.69 – 7.66 (m, 2H), 7.66 – 7.62 (m, 2H), 7.62 – 7.58 (m, 1H), 7.54 – 7.45 (m, 3H), 7.30 (d, *J* 8.1 Hz, 2H). ^{13}C NMR (101 MHz, $CDCl_3$) δ = 166.0, 149.1, 141.3, 139.8, 139.1, 137.7, 132.9, 130.0, 129.6, 128.8, 127.6, 126.8, 125.6, 124.6, 124.3, 123.9, 121.5. HRMS (ES+, *m/z*) calculated for $C_{20}H_{12}F_3NNaO_2Se$ $[M+Na]^+$ requires 457.9878. Found 457.9881. Elemental analysis calculated for $C_{20}H_{12}F_3NO_2Se$ requires, C, 55.31; H, 2.79; N, 3.23, found, C, 54.79; H, 2.71; N, 3.09. IR (neat): ν_{max}/cm^{-1}: 3088 (w), 3053 (w), 1597 (s), 1563 (s), 1483 (m), 1443 (m), 1269 (s, br), 1204 (s, br), 1154 (s, br). Melting Point:: 204 – 205 °C (EtOAc).

(4-((4-Fluoropiperidin-1-yl)methyl)phenyl)boronic acid

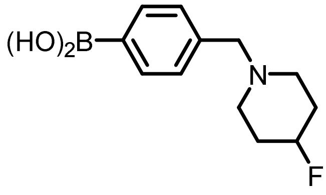

General procedure 4 was followed using (4-(bromomethyl)phenyl)boronic acid (0.52 g, 2.41 mmol, 1 eq) and 4-fluoropiperidine hydrochloride (0.36 g, 2.53 mmol, 1.05 eq) to give the title compound as a white foam (0.54 g, 95 %). ^{1}H NMR (400 MHz, $CDCl_3$) δ = 8.05 (s, 2H),

7.71 (d, *J* 7.8 Hz, 2H), 7.24 (d, *J* 7.8 Hz, 2H), 3.44 (s, 2H,), 3.38 – 3.12 (m, 2H), 2.45 (s, 1H), 2.25 (br s, 1H), 1.90 – 1.60 (m, 2H). ^{13}C NMR (101 MHz, $CDCl_3$) δ = 140.4, 134.3, 128.3, 88.8 (d, *J* 168.6 Hz), 62.2, 49.3, 31.3 (d, *J* 19.0 Hz). CI-HRMS: m/z calculated for $C_{12}H_{16}FN$ [M-(BO_2H)+H]$^+$ requires 194.1340. Found 194.1334, and 176.1436 [(M-BO_2H-F)+H]$^+$.

4'-((4-Fluoropiperidin-1-yl)methyl)-[1,1'-biphenyl]-3-amine

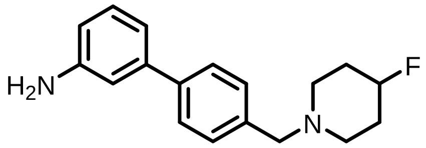

General procedure 3 was followed using (4-((4-fluoropiperidin-1-yl)methyl)phenyl)boronic acid. The crude mixture was purified by FCC (40 % EtOAc in Hex) to give 71d as a clear yellow oil (0.32 g, 50 %). ^{1}H NMR (400 MHz, $CDCl_3$) δ = 7.52 (d, *J* 8.1 Hz, 2H), 7.36 (d, *J* 8.1 Hz, 2H), 7.22 (t, *J* 7.8 Hz, 1H), 6.99 (d, *J* 7.7 Hz, 1H), 6.91 (apparent t, 1H), 6.67 (dd, *J* 7.9, 2.2 Hz, 1H), 4.79 – 4.59 (m, 1H), 3.54 (s, 2H), 2.68 – 2.55 (m, 2H), 2.46 – 2.36 (m, 2H), 2.00 – 1.81 (m, 4H). ^{13}C NMR (101 MHz, $CDCl_3$) δ = 146.9, 142.3, 140.3, 137.5, 129.8, 129.5, 127.1, 117.7, 114.2, 113.9, 88.9 (d, *J* 171.6 Hz), 62.8, 49.7 (d, *J* 5.8 Hz), 31.7 (d, *J* 19.4 Hz). HRMS (ES+, *m/z*) calculated for $C_{18}H_{22}FN_2$ [M+H]$^+$ requires 285.1762. Found 285.1772.

***N*-(4'-((4-fluoropiperidin-1-yl)methyl)-[1,1'-biphenyl]-3-yl)-2-iodobenzamide**

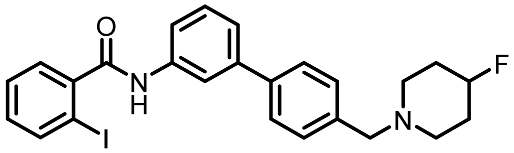

General procedure 1 was followed using 4'-((4-fluoropiperidin-1-yl)methyl)-[1,1'-biphenyl]-3-amine and 2-iodobenzoic acid. Note HCl not used in workup. The crude mixture was purified by FCC (100 % EtOAc) to give the title compound as a white solid (0.31 g, 53 %). ^{1}H NMR (400 MHz, $CDCl_3$) δ = 7.95 – 7.86 (m, 2H), 7.69 – 7.59 (m, 2H), 7.59 – 7.49 (m, 3H), 7.47 – 7.33 (m, 5H), 7.14 (t, *J* 7.4 Hz, 1H), 4.79 – 4.55 (m, 1H), 3.55 (s, 2H), 2.69 – 2.54 (m, 2H), 2.50 – 2.32 (m, 2H), 2.01 – 1.74 (m, 4H). ^{13}C NMR (101 MHz, $CDCl_3$) δ = 167.4, 142.2, 142.2, 140.2, 139.5, 138.1, 137.9, 131.7, 129.7, 128.7, 128.5, 127.2, 123.7,

119.0, 118.9, 92.5, 88.8 (d, *J* 170.1 Hz), 62.7, 49.6 (d, *J* 6.0 Hz), 31.6 (d, *J* 19.4 Hz). HRMS (ES+, *m/z*) calculated for $C_{25}H_{25}FIN_2O$ $[M+H]^+$ requires 515.0990. Found 515.0978.

2-(4'-((4-Fluoropiperidin-1-yl)methyl)-[1,1'-biphenyl]-3-yl)benzo[*d*][1,2]selenazol-3(2*H*)-one (8)

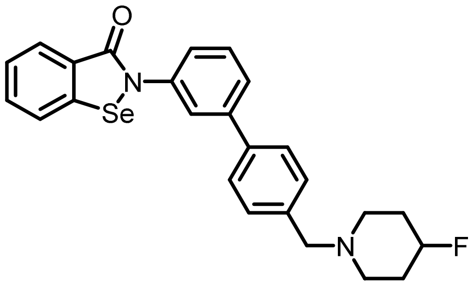

General procedure 2 was followed using *N*-(4'-((4-fluoropiperidin-1-yl)methyl)-[1,1'-biphenyl]-3-yl)-2-iodobenzamide. The crude product was purified by FCC (100 % EtOAc) and the residue was recrystallised from ethyl acetate to give the title compound as a white solid (0.030 g, 11 %). ^{1}H NMR (400 MHz, $CDCl_3$) δ = 8.14 (d, *J* 7.8 Hz, 1H), 7.87 (s, 1H), 7.70 – 7.63 (m, 2H), 7.62 – 7.54 (m, 3,), 7.53 – 7.45 (m, 3H,), 7.40 (d, *J* 8.0 Hz, 2H), 4.81 – 4.57 (m, 1H), 3.55 (s, 2H), 2.69 – 2.56 (m, 2H), 2.45 – 2.32 (m, 2H), 2.00 – 1.83 (m, 4H). ^{13}C NMR (101 MHz, $CDCl_3$) δ = 165.9, 142.5, 139.7, 139.1, 138.3, 137.8, 132.8, 129.8, 129.7, 129.6, 127.7, 127.3, 126.8, 125.6, 124.3, 124.2, 123.9, 88.9 (d, *J* 169.9 Hz), 62.8, 49.7 (d, *J* 6.0 Hz), 31.7 (d, *J* 19.4 Hz). HRMS (ES+, *m/z*) calculated for $C_{25}H_{23}FN_2OSe$ $[M+H]^+$ requires 467.1032. Found 467.1028. Elemental analysis calculated for $C_{25}H_{23}FN_2OSe$ requires C, 64.52; H, 4.98; F, 4.08; N, 6.02, found C, 64.70; H, 4.90; N, 5.97. Melting Point:: 181 – 182 °C (EtOAc).

(4-(Morpholinomethyl)phenyl)boronic acid

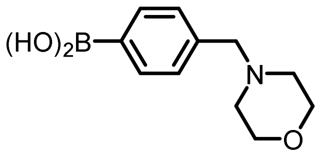

General procedure 4 was followed using (4-(bromomethyl)phenyl)boronic acid and morpholine to give the title compound as an off white solid (0.91 g, 87 %). ^{1}H NMR (400 MHz, $CDCl_3$) δ = 8.07 – 7.68 (m, 2H), 7.29 (d, *J* 6.9 Hz, 2H), 3.77 – 3.65 (m, 4H), 3.53 (s, 2H), 2.46 (s, 4H). ^{13}C NMR (101 MHz, $CDCl_3$) δ = 134.1, 128.6, 67.0, 63.7, 53.7. HRMS (CI, *m/z*) calculated for $C_{11}H_{15}NO$ [M-(BO_2H)+H]$^+$ requires 178.1226. Found 178.1232.

4'-(morpholinomethyl)-[1,1'-biphenyl]-3-amine

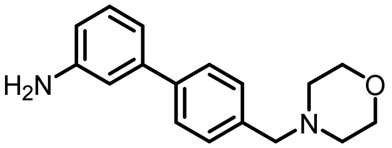

General procedure 3 was followed using (4-(morpholinomethyl)phenyl)boronic acid. The crude mixture was purified by FCC (40 % EtOAc in Hex) to give the title compound as a yellow to orange oil (0.98 g, 90 %). ^{1}H NMR (400 MHz, $CDCl_3$) δ = 7.52 (d, *J* 8.1 Hz, 2H), 7.37 (d, *J* 8.1 Hz, 2H), 7.22 (apparent t, *J* 7.8 Hz, 1H), 6.99 (d, *J* 7.7 Hz, 1H), 6.90 (apparent t, 1H), 6.67 (dd, *J* 7.9, 1.6 Hz, 1H), 3.76 – 3.70 (apparent t, 4H), 3.53 (s, 2H), 2.48 (br s, 4H). ^{13}C NMR (101 MHz, $CDCl_3$) δ = 146.8, 142.2, 140.4, 136.8, 129.8, 129.7, 127.1, 117.7, 114.2, 113.9, 67.1, 63.3, 53.8. HRMS (ES+, *m/z*) calculated for $C_{17}H_{21}N_2O$ [M+H]$^+$ requires 269.1648. Found 269.1647.

2-iodo-*N*-(4'-(morpholinomethyl)-[1,1'-biphenyl]-3-yl)benzamide

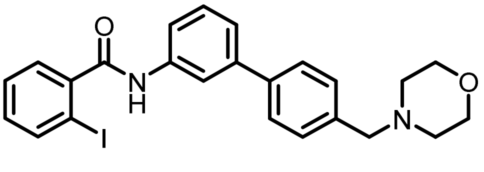

General procedure 1 was followed using 4'-(morpholinomethyl)-[1,1'-biphenyl]-3-amine and 2-iodobenzoic acid. Note HCl not used in workup. The crude mixture was purified by FCC (50 % EtOAc in Hex) to give the title compound as a white solid (1.15 g, 58 %). ^{1}H NMR (400 MHz, $CDCl_3$) δ = 7.94 – 7.88 (m, 2H), 7.66 – 7.59 (m, 2H), 7.59 – 7.52 (m, 3H), 7.47 – 7.38 (m, 5H), 7.15 (apparent td, 1H), 3.75 – 3.69 (apparent t, 4H), 3.54 (s, 2H), 2.51 – 2.44 (apparent t, 4H). ^{13}C NMR (101 MHz, $CDCl_3$) δ = 167.4, 142.3, 142.2, 140.2, 139.6, 138.1,

137.4, 131.7, 129.8, 129.7, 128.7, 128.5, 127.3, 123.7, 119.0, 118.9, 92.5, 67.2, 63.2, 53.8. HRMS (ES+, *m/z*) calculated for $C_{24}H_{27}IN_2O_2$ $[M+H]^+$ requires 499.0877. Found 499.0877.

2-(4'-(morpholinomethyl)-[1,1'-biphenyl]-3-yl)benzo[*d*][1,2]selenazol-3(2*H*)-one (9)

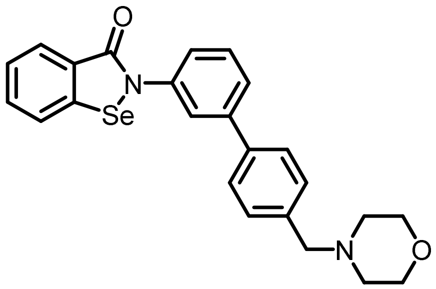

General procedure 2 was followed using 2-iodo-*N*-(4'-(morpholinomethyl)-[1,1'-biphenyl]-3-yl)benzamide. The crude product was purified by FCC (50 % EtOAc in hexane) and the residue was recrystallised from ethyl acetate to give the title compound as a white solid (0.094 g, 18 %). ^{1}H NMR (400 MHz, $CDCl_3$) δ = 8.14 (d, *J* 7.8 Hz, 1H), 7.87 (s, 1H), 7.71 – 7.63 (m, 2H), 7.61 – 7.54 (m, 3H), 7.53 – 7.46 (m, 3H), 7.41 (d, *J* 8.0 Hz, 2H), 3.78 – 3.69 (apparent t, 4H), 3.54 (s, 2H), 2.48 (br s, 4H). ^{13}C NMR (101 MHz, $CDCl_3$) δ = 165.9, 142.5, 139.7, 139.3, 137.8, 137.6, 132.8, 129.83, 129.81, 129.6, 127.7, 127.3, 126.8, 125.6, 124.3, 124.2, 123.9, 67.2, 63.2, 53.8. HRMS (ES+, *m/z*) calculated for $C_{24}H_{23}N_2O_2Se$ $[M+H]^+$ requires 451.0919. Found 451.0906. Elemental analysis calculated for $C_{24}H_{22}N_2O_2Se$ requires, C, 64.14; H, 4.93; N, 6.23, found C, 63.73; H, 4.82; N, 6.09. IR (neat): ν_{max}/cm^{-1}: 3088 (w), 3053 (w), 2856 (w), 2800 (w), 1587 (s), 1561 (s), 1441 (m), 1307 (s), 1108 (s). Melting Point:: 179 – 180 °C (EtOAc).

(4-((4-methylpiperazin-1-yl)methyl)phenyl)boronic acid

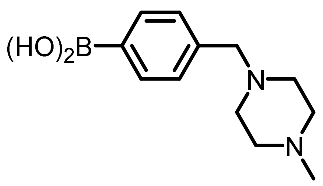

General procedure 4 was followed using (4-(bromomethyl)phenyl)boronic acid and *N*-methylpiperazine to give the title compound as a white solid (0.51 g, 46 %). ^{1}H NMR (400 MHz, MeOD) δ = 7.60 (d, *J* 6.9 Hz, 2H), 7.30 (d, *J* 7.8 Hz, 2H), 3.57 (s, 1H), 2.57 (s, 8H), 2.34 (s, 3H). ^{13}C NMR (101 MHz, DMSO) δ = 140.5, 140.2, 134.1, 133.9, 128.0, 127.9, 62.2, 54.7, 52.6, 45.7. HRMS (CI, *m/z*) calculated for $C_{12}H_{19}N_2$ [M-(BO_2H)+H]$^+$ requires 191.1543. Found 191.1547.

4'-((4-methylpiperazin-1-yl)methyl)-[1,1'-biphenyl]-3-amine

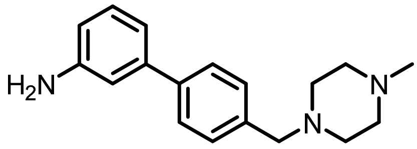

General procedure 3 was followed using (4-((4-methylpiperazin-1-yl)methyl)phenyl)boronic acid. The crude mixture was purified by FCC (5 % MeOH in EtOAc) to give the product as a clear orange oil (1.01 g, 94 %). ^{1}H NMR (400 MHz, $CDCl_3$) δ = 7.50 (d, *J* 8.1 Hz, 2H), 7.36 (d, *J* 8.1 Hz, 2H), 7.21 (apparent t, *J* 7.8 Hz, 1H), 6.98 (d, *J* 7.7 Hz, 1H), 6.90 (apparent t, 1H), 6.67 (dd, *J* 7.8, 1.8 Hz, 1H), 3.73 (br s, 2H), 3.54 (s, 2H), 2.48 (s, *J* 11.3 Hz, 8H), 2.29 (s, 3H). ^{13}C NMR (101 MHz, $CDCl_3$) δ = 146.8, 142.3, 140.3, 137.3, 129.8, 129.6, 127.0, 117.7, 114.1, 113.9, 62.9, 55.3, 53.2, 46.2. HRMS (ES+, *m/z*) calculated for $C_{18}H_{24}N_3$ [M+H]$^+$ requires 282.1965. Found 282.1969.

2-iodo-*N*-(4'-((4-methylpiperazin-1-yl)methyl)-[1,1'-biphenyl]-3-yl)benzamide

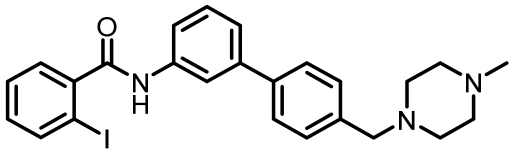

General procedure 1 was followed using 4'-((4-methylpiperazin-1-yl)methyl)-[1,1'-biphenyl]-3-amine and 2-iodobenzoic acid. Note HCl not used in workup. The crude mixture was purified by FCC (10 % MeOH in EtOAc and a few drops of NEt_3) to give the title compound

as a sticky off white to light yellow foam (1.00 g, 74 %). ^{1}H NMR (400 MHz, $CDCl_3$) δ = 7.92 (d, *J* 7.9 Hz, 1H), 7.87 (s, 1H), 7.66 – 7.60 (m, 2H), 7.58 – 7.53 (m, 3H), 7.48 – 7.42 (m, 2H), 7.42 – 7.37 (m, 3H), 7.16 (apparent td, 1H), 3.55 (s, 2H), 2.51 (s, 8H), 2.31 (s, 3H). ^{13}C NMR (101 MHz, $CDCl_3$) δ = 167.4, 142.2, 142.2, 140.2, 139.5, 138.1, 137.7, 131.7, 129.8, 129.7, 128.7, 128.5, 127.2, 123.8, 119.0, 118.9, 92.5, 62.8, 55.2, 53.1, 46.1. HRMS (ES+, *m/z*) calculated for $C_{25}H_{26}IN_3O$ [M+H]$^+$ requires 512.1193. Found 512.1197.

2-(4'-((4-methylpiperazin-1-yl)methyl)-[1,1'-biphenyl]-3-yl)benzo[*d*][1,2]selenazol-3(2*H*)-one (10)

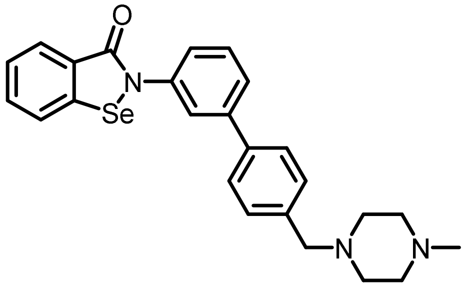

General procedure 2 was followed using 2-iodo-*N*-(4'-((4-methylpiperazin-1-yl)methyl)-[1,1'-biphenyl]-3-yl)benzamide. The crude product was purified by FCC (20 % MeOH in EtOAc with a few drops of NEt_3) to give the title compound as a yellow to off white foam (0.012 g, 7 %). ^{1}H NMR (400 MHz, $CDCl_3$) δ = 8.13 (d, *J* 7.8, 1H), 7.86 (s, 1H), 7.72 – 7.64 (m, 2H), 7.61 – 7.54 (m, 3H), 7.52 – 7.46 (m, 3H), 7.39 (d, *J* 8.0 Hz, 2H), 3.57 (s, 2H), 2.60 (br s, 8H), 2.38 (s, 3H). ^{13}C NMR (101 MHz, $CDCl_3$) δ = 165.9, 142.4, 139.7, 139.2, 137.83, 137.81, 132.7, 129.8, 129.5, 127.7, 127.2, 126.7, 125.5, 124.3, 124.2, 124.0, 62.7, 55.1, 52.9, 46.0. HRMS (ES+, *m/z*) calculated for $C_{25}H_{26}N_3OSe$ [M+H]$^+$ requires 464.1236. Found 464.1243. Elemental analysis calculated for $C_{25}H_{25}N_3OSe$ requires, C, 64.93; H, 5.45; N, 9.09, found, C, 61.78; H, 5.25; N, 8.60.

2-(3-Oxobenzo[d]isothiazol-2(3H)-yl)acetic acid

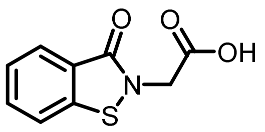

To a solution of 1,2-benzoisothiazol-3(2H)-one (1.0 eq) in THF (15 ml), NaH (1.2 eq) was added in portions while the reaction was stirred at 0 °C for 15 minutes. The solvent was then removed and 2,2,2-trifluoroethanol added at 0 °C, along with *tert*-butylbromoacetate (1.0 eq) and the reaction refluxed for 1 hour. The solvent was then removed and the reaction diluted with ethyl acetate, acidified with 1 M HCl, basified with 1 M NaOH, washed with brine, dried over $MgSO_4$ and concentrated to give the crude material as a pink solid. To the solid was added dichloromethane (10 ml) and trifluoroacetic acid (20 ml) and the reaction stirred overnight at room temperature. The reaction was then evaporated to dryness and washed with ethyl acetate (3 x 20 ml), to give the title compound (3.58 g, 52% yield) as a white solid. No further purification was required. ^{1}H NMR (400 MHz, $CDCl_3$) δ 13.06 (bs, 1H), 7.99 (d, *J* = 8.0 Hz, 1H), 7.89 (d, *J* = 8.0 Hz, 1H), 7.71 (t, *J* = 7.4 Hz, 1H), 7.45 (t, *J* = 7.4 Hz, 1H). ^{13}C NMR (100 MHz, $CDCl_3$) δ 169.79, 165.36, 141.77, 132.54, 126.12, 125.93, 122.77, 122.33, 44.73. HRMS (CI+, *m/z*) Calculated for $C_9H_7NO_3S$: 164.0165. Found $[M+H]^+$: 164.0167 (Diff -1.67 ppm).

tert-Butyl 2-(3-oxobenzo[d]isothiazol-2(3H)-yl)acetate (12)

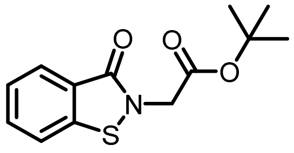

^{1}H NMR (400 MHz, $CDCl_3$) δ 8.05 (d, 1H, *J* = 8.1 Hz), 7.62 (dd, 1H, *J* = 8.2 & 7.0 Hz), 7.55 (d, 1H, *J* = 8.2 Hz), 7.40 (dd, 1H, *J* = 8.1 & 7.0 Hz), 4.51 (s, 2H), 1.49 (s, 9H). ^{13}C NMR (100 MHz, $CDCl_3$) δ 166.7, 165.8, 140.9, 132.2, 126.9, 125.5, 123.6, 120.3, 83.0, 45.3, 28.0. HRMS (ES+, *m/z*) Calculated for $C_{13}H_{15}NO_3NaS$: 288.0670. Found $[M+H]^+$: 288.0668 (Diff -1.67 ppm). Elemental analysis calculated for $C_{13}H_{15}NO_3$ requires C, 58.85; H, 5.70; N, 5.28. Found C, 58.69; H, 5.64; N, 5.15. IR (neat): ν_{max}/cm^{-1}: (solid) 2998 (m), 2982 (m), 2935 (m), 1740 (s), 1660 (s), 1624 (s), 1459 (m), 1257 (m). Melting Point:: 68 - 70 °C.

***N*-(4-(4-Fluorophenoxy)phenyl)-2-(3-oxobenzo[d]isothiazol-2(3H)-yl)acetamide (13)**

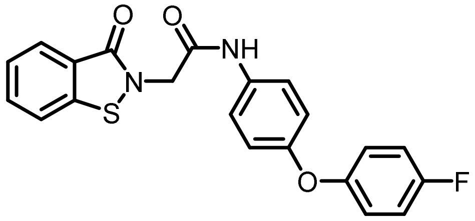

General procedure 5 was followed using 4-(4-fluorophenoxy)aniline. The crude product was purified by FCC (20 % EtOAc in Hex) to give the title compound as a white solid (0.075 g, 13% yield). ^{1}H NMR (400 MHz, $CDCl_3$) δ 8.76 (s, 1H), 8.05 (d, *J* = 7.9 Hz, 1H), 7.70 – 7.68 (m, 1H), 7.65 (d, *J* = 8.1 Hz, 1H), 7.49 – 7.45 (m, 3H), 7.03 – 7.00 (m, 2H), 6.96 – 6.91 (m, 4H), 4.63 (s, 2H). ^{13}C NMR (100 MHz, $CDCl_3$) δ 166.5, 165.1, 154.2, 141.1, 132.8, 132.7, 126.8, 126.0, 123.2, 121.8, 120.5, 120.1, 120.0, 119.0, 116.4, 116.1, 49.2. HRMS (ES+, *m/z*) Calculated for $C_{21}H_{15}N_2O_3FS^{23}Na$: 417.0685. Found $[M+Na]^+$: 417.0673 (Diff – 2.88 ppm). Elemental analysis calculated for $C_{21}H_{15}N_2O_3SF$ requires C, 63.95; H, 3.83; N, 7.10. Found C, 64.17; H, 4.13; N, 6.79. IR (neat): ν_{max}/cm^{-1}: (solid) 3267 (m), 3076 (m), 2924 (m), 1686 (s), 1631 (d), 1495 (m), 1407 (m), 1290 (m). Melting Point: 202-204 °C.

2-(1-Oxoisoindolin-2-yl)-N-(4-phenoxyphenyl)acetamide (14)

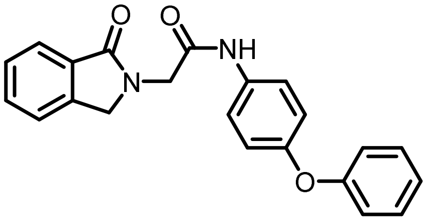

General procedure 6 was followed using 2-(1-oxoisoindolin-2-yl)acetic acid and 4-phenoxyaniline. The crude product was purified by trituration in hexane to obtain the title compound as a white solid (0.12 g, 43 % yield). ^{1}H NMR (400 MHz, DMSO) δ 10.23 (s, 1H), 7.72 (d, *J* = 7.4 Hz, 1H), 7.62 (d, *J* = 7.8 Hz, 1H), 7.61 (d, *J* = 8.9 Hz, 1H), 7.55 – 7.50 (m, 1H), 7.37 (dd, *J* = 8.1, 7.9 Hz, 2H), 7.10 (t, *J* = 7.4 Hz, 1H), 6.99 (d, *J* = 8.9 Hz, 2H), 6.96 (d, *J* = 8.1 Hz, 2H), 4.59 (s, 2H), 4.40 (s, 2H). ^{13}C NMR (100 MHz, DMSO) δ 168.4, 167.1, 157.7, 152.4, 142.8 135.1, 132.4, 132.0, 130.4, 128.3, 124.0, 123.5, 123.3, 121.4, 119.9, 118.4, 51.1, 45.7. HRMS (ES+, *m/z*) Calculated for $C_{22}H_{18}N_2O_3^{23}Na$: 381.1215. Found $[M+Na]^+$: 381.1208 (Diff -1.84 ppm). Elemental analysis calculated for $C_{22}H_{18}N_2O_3$ requires C, 73.73; H, 5.06%; N 7.82. Found C, 73.36; H, 5.00; N, 7.69. IR (neat): ν_{max}/cm^{-1}: (solid)

3306 (s), 3042 (m), 2959 (m), 1666 (s). 1617 (s), 1455 (m), 1228 (m). Melting Point: 185 – 187 °C.

2-(1,3-Dioxoisoindolin-2-yl)-N-(4-phenoxyphenyl)acetamide (15)

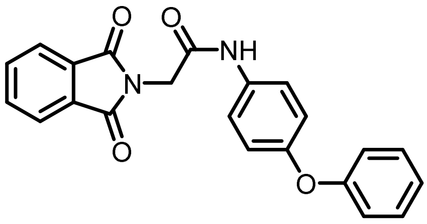

General procedure 6 was followed using 2-(1,3-dioxoisoindolin-2-yl)acetic acid and 4-phenoxyaniline. The crude product was purified by trituration in hexane to obtain the title compound as a white solid (0.16 g, 59% yield). ^{1}H NMR (400 MHz, DMSO) δ 10.35 (s, 1H), 7.92 (m, 4H), 7.57 (d, *J* = 8.3 Hz, 2H), 7.37 (app. t, *J* = 8.3, 7.9 Hz, 2H), 7.10 (app. t, *J* = 7.9 Hz, 1H), 6.99 (d, *J* = 8.6 Hz, 2H), 6.97 (d, *J* = 8.6 Hz, 2H), 4.44 (s, 2H). ^{13}C NMR (100 MHz, DMSO) δ 168.0, 165.1, 157.7, 152.6, 135.2, 134.8, 132.1, 130.4, 123.8, 128.5, 121.4 119.9 118.5, 41.2. HRMS (CI+, *m/z*) Calculated for $C_{22}H_{17}N_2O_4$: 373.1183. Found $[M+H]^+$: 373.1190 (Diff -3.85 ppm). IR (neat): ν_{max}/cm^{-1}: (solid) 3261 (s). 2979 (m), 2889 (m), 1775 (s), 1723 (s), 1658 (s), 1464 (m), 1266 (m). Melting Point: 201 – 203 °C. Purity HPLC 95.1%, R_t = 10.41 min.

2-(4-Chlorobenzyl)benzo[d]isothiazol-3(2H)-one (16)

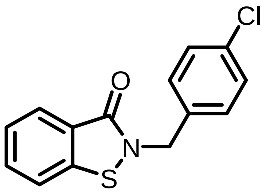

General procedure 7 was followed using 4-chlorobenzyl bromide. The crude product was purified by FCC (12.5 % EtOAc in Hex) to give the title compound as a yellow solid (0.17 g, 47% yield). ^{1}H NMR (400 MHz, $CDCl_3$) δ 8.06 (d, *J* = 7.9 Hz, 1H), 7.60 – 7.58 (m, 1H), 7.50 (d, *J* = 7.9 Hz, 1H), 7.41 – 7.39 (m, 1H), 7.33 – 7.28 (m, 4H), 5.01 (s, 2H). ^{13}C NMR (100 MHz, $CDCl_3$) δ 165.4, 140.3, 134.7, 134.2, 132.0, 129.8, 129.0, 126.9, 125.6, 124.3, 120.5, 46.8. HRMS (CI+, *m/z*) Calculated for $C_{14}H_{11}ClNOS$: 276.0244. Found $[M+H]^+$:

276.0250 (Diff -2.09 ppm). Elemental analysis calculated for $C_{14}H_{10}ClNOS$ requires C, 60.98; H, 3.66; N, 5.08. Found C, 60.81; H, 3.63; N, 4.85. IR (neat): ν_{max}/cm^{-1}: (solid) 3056 (m), 928 (m), 1655 (s), 1459 (m), 1285 (m), 735 (s). Melting Point: 64 - 66 °C.

2-(4-Bromobenzyl)benzo[d]isothiazol-3(2H)-one (17)

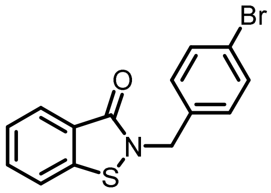

General procedure 7 was followed using 4-bromobenzyl bromide. The crude product was purified by FCC (12.5 % EtOAc in Hex) to give the title compound as a yellow solid (0.60 g, 56% yield). ^{1}H NMR (400 MHz, $CDCl_3$) δ 8.06 (d, *J* = 7.9 Hz, 1H), 7.60 (dd, *J* = 7.2, 8.1 Hz, 1H), 7.50 (d, *J* = 8.1 Hz, 1H), 7.47 (d, *J* = 8.4 Hz, 2H), 7.41 (app. t, *J* = 7.2, 7.9 Hz, 1H), 7.22 (d, *J* = 8.4 Hz, 2H), 5.00 (s, 2H). ^{13}C NMR (100 MHz, $CDCl_3$) δ 165.4, 140.3, 135.2, 132.1, 132.0, 130.1, 126.9, 125.7, 124.3, 122.4, 120.5, 46.1. HRMS (CI+, *m/z*) Calculated for $C_{14}H_{11}BrNOS$: 319.9739. Found $[M+H]^+$: 319.9739 (Diff 0.16 ppm). Elemental analysis calculated for $C_{14}H_{10}BrNOS$ requires C, 52.51; H, 3.15; N, 4.37. Found C, 51.86; H 3.09; N 4.17. IR (neat): ν_{max}/cm^{-1}: (solid) 2978 (m), 2928 (m), 1657 (s), 1446 (m), 1234 (m), 736 (m). Melting Point: 103 – 105 °C.

2-(2-Fluorobenzyl)benzo[d]isothiazol-3(2H)-one (18)

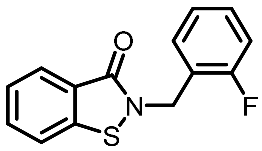

General procedure 7 was followed using 2-fluorobenzyl bromide. The crude product was purified by FCC (12.5 % EtOAc in Hex) to give the title compound as a pale yellow solid (0.085 g, 25% yield). ^{1}H NMR (400 MHz, $CDCl_3$) δ 7.98 (d, *J* = 7.8 Hz, 1H), 7.51 (m, 1H), 7.42 (d, *J* = 7.8 Hz, 1H), 7.31 (m, 2H), 7.21 (m, 1H), 7.02 (m, 2H), 5.05 (s, 2H). ^{13}C NMR (100 MHz, $CDCl_3$) δ 165.4, 160.8 (d, *J* = 247. 8 Hz), 140.5, 131.9, 130.8 (d, *J* = 3.5 Hz), 130.2 (d, *J* = 8.2 Hz), 126.8, 125.5, 124.6 (d, *J* = 3.4 Hz), 124.3, 123.3 (d, *J* = 14.6 Hz), 102.4, 115.5 (d, *J* = 21.3 Hz), 41.0. HRMS (CI+, *m/z*) Calculated for $C_{14}H_{11}FNOS$:

260.0540. Found $[M+H]^+$: 260.0544 (Diff – 1.52 ppm). IR (neat): ν_{max}/cm^{-1}: (solid) 3056 (m), 3015 (m), 2932 (m), 1652 (s), 1488 (m), 1356 (s). Melting point 98 -100 °C. Purity HPLC 97.6%, R_t = 9.55 min.

2-(2-(4-Fluoropiperidin-1-yl)-2-oxoethyl)benzo[d]isothiazol-3(2H)-one (19)

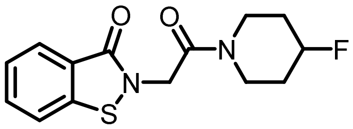

General procedure 5 was followed using 4-fluoropiperdine hydrochloride. The crude product was purified by FCC (20 % EtOAc in Hex) to give the title compound as a white solid (0.13 g, 19% yield). ^{1}H NMR (400 MHz, $CDCl_3$) δ 8.04 (d, *J* = 7.9 Hz, 1H), 7.63 (app. t, *J* = 8.1 Hz, 1H), 7.56 (d, *J* = 7.9 Hz, 1H), 7.40 (app. t, *J* = 8.1 Hz, 1H), 4.97 – 4.80 (m, 1H), 4.62 (d, *J* = 16.0 Hz, 1H), 3.97 – 3.95 (m, 1H), 3.65 – 3.63 (m, 2H), 3.48 – 3.47 (m, 1H), 1.88 – 1.84 (m, 4H). ^{13}C NMR (100 MHz, $CDCl_3$) δ 165.7, 165.0, 141.4, 132.2, 126.8, 125.5, 123.5, 120.4, 87.1 (d, *J* = 172.2 Hz), 45.0, 41.3 (d, *J* = 4.9 Hz), 38.2 (d, *J* = 4.2 Hz), 31.4 (d, *J* = 18.6 Hz), 30.9 (d, *J* = 17.7 Hz). HRMS (CI+, *m/z*) Calculated for $C_{14}H_{15}FN_2O_2S$: 295.0911. Found $[M+H]^+$: 295.0917 (Diff -1.99 ppm). Elemental analysis calculated for $C_{14}H_{15}FN_2O_2S$ requires C, 57.13; H, 5.14; N, 9.52. Found C, 56.82; H, 5.02; N, 9.44. IR (neat): ν_{max}/cm^{-1}: (solid) 2974 (m), 2937 (m), 1637 (s), 1596 (s), 1450 (m), 1252 (m). Melting Point: 94 – 97 °C. Purity HPLC 90.5%, R_t = 7.88 min.

***N*-(4-fluorobenzyl)-*N*-methyl-2-(3-oxobenzo[d]isothiazol-2(3H)-yl)acetamide (20)**

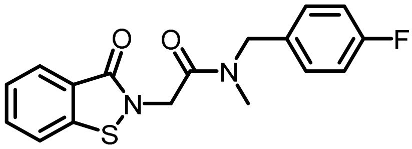

General procedure 5 was followed using 1-(4-fluorophenyl)-*N*-methylmethanamine. The crude product was purified by FCC (20 % EtOAc in Hex) to give the title compound as a yellow oil (52.95%). ^{1}H NMR (400 MHz, DMSO) exists as a mixture of two rotamers 1:2. δ 7.97 (m, 1H), 7.91 (m,1H), 7.87 (m, 1H), 7.71 (app. T, 1H), 7.47 – 7.16 (m, 5H), 4.83 (m, 2H), 4.80 (s, 2H), 4.51 (d, 2H), 4.67 (d, 2H), 3.01 - 2.48 (m, 3H). ^{13}C NMR (100MHz, DMSO) δ 167.0, 165.6, 165.4, 141.9, 137.8, 132.4, 130.1, 130.2, 130.0, 127.3, 124.0, 122.2,

115.8, 115.6, 60.2, 50.9, 34.3. HRMS (ES+, *m/z*) Calculated for $C_{17}H_{15}FN_2O_2SNa$ 353.3666. Found $[M+Na]^+$: 353.0728.

References

1 Wojtowicz, H. *et al.* Azaanalogues of ebselen as antimicrobial and antiviral agents: synthesis and properties. *Farmaco (Societa chimica italiana : 1989)* 59, 863-868, doi:10.1016/j.farmac.2004.07.003 (2004).

2 Yao, B., Wang, Q. & Zhu, J. Palladium(II)-catalyzed cyclizative cross-coupling of ortho-alkynylanilines with ortho-alkynylbenzamides under aerobic conditions. *Angewandte Chemie (International ed. in English)* 52, 12992-12996, doi:10.1002/anie.201307738 (2013).

3 Balkrishna, S. J., Bhakuni, B. S., Chopra, D. & Kumar, S. Cu-catalyzed efficient synthetic methodology for ebselen and related Se-N heterocycles. *Organic letters* 12, 5394-5397, doi:10.1021/ol102027j (2010).

4 Khan, M. W. & Reza, A. F. G. M. Palladium mediated synthesis of isoindolinones and isoquinolinones. *Tetrahedron* 61, 11204-11210, doi:https://doi.org/10.1016/j.tet.2005.09.032 (2005).

5 Chang, T. C., Huang, M. L., Hsu, W. L., Hwang, J. M. & Hsu, L. Y. Synthesis and biological evaluation of ebselen and its acyclic derivatives. *Chemical & pharmaceutical bulletin* 51, 1413-1416, doi:10.1248/cpb.51.1413 (2003).
