# Supplementary material for: Ebselen as template for stabilization of A4V mutant dimer for motor neuron disease therapy
Source: Commun Biol. 2020 Mar 5;3:97. doi: 10.1038/s42003-020-0826-3 (PMC7058017; doi:10.1038/s42003-020-0826-3)
Supplement: Supplementary file 2 — Description of Additional Supplementary Files [file 42003_2020_826_MOESM2_ESM.pdf]

DSF data files for (a) Wild type SOD1 with Ebselen series compounds, (b) A4V SOD1 with Ebselen series compounds, (c) C6S SOD1 with Ebselen series compounds, (d) A4VC6S with Ebselen compound series (e) Wild type SOD with ebsulphur, (f) A4V SOD1 with Ebsulphur, (g) C6S SOD1 with Ebsulphur compound series and (h)A4V C6S SOD1 with Ebsulphur compound series
